# Retraction statement: Hyaluronic acid‐rich burger separator edible disc prepared from slaughterhouse waste

**DOI:** 10.1002/fsn3.3263

**Published:** 2023-02-26

## Abstract

Kalantarmahdavi, M., Salari, A., Pasdar, Z., & Amiryousefi, M. R. (2022). Hyaluronic acid‐rich burger separator edible disc prepared from slaughterhouse waste. *Food Science & Nutrition, 10*, 3562–3573. https://doi.org/10.1002/fsn3.2740.

The above article from *Food Science & Nutrition*, published online on 20 January 2022 in Wiley Online Library (wileyonlinelibrary.com), has been retracted by agreement between the authors, journal's editor‐in‐chief, Y. Martin Lo, and Wiley Periodicals LLC. This action has been agreed due to an error at the publishers which caused a duplicate of the article to be published on 20 June 2022. The correct version of the article is to be found at ‘Edible hyaluronic acid‐rich burger separator discs prepared from slaughterhouse waste’ by Mahboubeh Kalantarmahdavi, Amir Salari, Zahra Pasdar, Mohamad Reza Amiryousefi (https://doi.org/10.1002/fsn3.2954).

Kalantarmahdavi, M., Salari, A., Pasdar, Z., & Amiryousefi, M. R. (2022). Hyaluronic acid‐rich burger separator edible disc prepared from slaughterhouse waste. *Food Science & Nutrition, 10*, 3562–3573. https://doi.org/10.1002/fsn3.2740.

The above article from *Food Science & Nutrition*, published online on 20 January 2022 in Wiley Online Library (wileyonlinelibrary.com), has been retracted by agreement between the authors, journal's editor‐in‐chief, Y. Martin Lo, and Wiley Periodicals LLC. This action has been agreed due to an error at the publishers which caused a duplicate of the article to be published on 20 June 2022. The correct version of the article is to be found at ‘Edible hyaluronic acid‐rich burger separator discs prepared from slaughterhouse waste’ by Mahboubeh Kalantarmahdavi, Amir Salari, Zahra Pasdar, Mohamad Reza Amiryousefi (https://doi.org/10.1002/fsn3.2954).

